# Venarsen in Tertiary Syphilis

**Published:** 1915-09

**Authors:** M. T. Davis

**Affiliations:** Atlanta, Ga.


### VENARSEN IN TERTIARY SYPHILIS.

By M. T. Davis, M. D., Atlanta, Ga.

Owing to the scarsity of Salvarsan and Neosalvarsan, and the extortionate charges being made for these products in certain quarters, I have been led to test the merits of Venarsen, in several cases that have come under my observation recently. One of these, it is my privilege to present to-night, the results in the case being such as to justify the belief that, at least in Tertiary Syphilis, we have in Venarsen a remedy of considerable value.

This patient came to me complaining of pains in the head, back and limbs, all worse at night. An examination revealed a varicosed condition of the veins of both legs, the varicosity being as marked as any I have seen in recent years. The veins had ruptured at several points, resulting in extensive ulceration, the condition having existed for fifteen years, the bone being exposed in several places.

The patient denies a specific history, but admits several miscarriages. A Wassermann was not made, but the Luetin skin test, which I did make, was strongly positive.

Venarsen was given at five-day intervals, for three doses, at which time the ulcerating surfaces had completely healed, with entire subsidence of all pain. Treatment was continued at the same intervals for four more doses, making seven in all, leaving the patient in the improved condition so evident to-night. Each time I gave Venarsen, I also administered intravenously one eighth grain of oxycyanide of Mercury, which is now supplied in ampoules by the manufacturers of Venarsen.

According to a written report received by Drs. Merritt and Aven, the manufacturers state that this product consists of Sodium Cocodylate, and Biniodide of Mercury. Since there is only one fortieth grain of the Mercury in each dose, I am of the opinion that its therapeutic effects are almost negligible,—hence my reason for giving additional Mercury in the form of the oxycyanide.

I would not be understood as advocating Venarsen to the exclusion of Salvarsan and Neosalvarsan, nor do I believe that it will ever take the place of these tried and proven remedies of value. But inasmuch as these products are at times difficult to obtain, and just now very expensive, I do believe that in appropriate cases we are justified in giving Venarsen a fair and impartial trial.

